# Evaluating the potential of endothelial cells derived from human induced pluripotent stem cells to form microvascular networks in 3D cultures

**DOI:** 10.1038/s41598-018-20966-1

**Published:** 2018-02-08

**Authors:** Jonathan R. Bezenah, Yen P. Kong, Andrew J. Putnam

**Affiliations:** 1 Department of Chemical Engineering, University of Michigan, Ann Arbor, Michigan 48109 USA; 2 Department of Biomedical Engineering, University of Michigan, Ann Arbor, Michigan 48109 USA

## Abstract

A major translational challenge in the fields of therapeutic angiogenesis and regenerative medicine is the need to create functional microvasculature. The purpose of this study was to assess whether a potentially autologous endothelial cell (EC) source derived from human induced pluripotent stem cells (iPSC-ECs) can form the same robust, stable microvasculature as previously documented for other sources of ECs. We utilized a well-established *in vitro* assay, in which endothelial cell-coated (iPSC-EC or HUVEC) beads were co-embedded with fibroblasts in a 3D fibrin matrix to assess their ability to form stable microvessels. iPSC-ECs exhibited a five-fold reduction in capillary network formation compared to HUVECs. Increasing matrix density reduced sprouting, although this effect was attenuated by distributing the NHLFs throughout the matrix. Inhibition of both MMP- and plasmin-mediated fibrinolysis was required to completely block sprouting of both HUVECs and iPSC-ECs. Further analysis revealed MMP-9 expression and activity were significantly lower in iPSC-EC/NHLF co-cultures than in HUVEC/NHLF co-cultures at later time points, which may account for the observed deficiencies in angiogenic sprouting of the iPSC-ECs. Collectively, these findings suggest fundamental differences in EC phenotypes must be better understood to enable the promise and potential of iPSC-ECs for clinical translation to be realized.

## Introduction

Numerous cardiovascular diseases are characterized by ischemia, a reduction/obstruction of oxygenated blood supply to tissues, which can eventually lead to necrosis^1^. Due to the increasing number of deaths and costs attributed to ischemic diseases, it is critical to create new therapies focused on rebuilding vasculature to provide cells with sufficient oxygen and nutrients to prevent additional necrosis and amputations^2–4^.

Over the past decade, several therapeutic approaches have emerged to promote angiogenesis and vasculogenesis, the processes by which new blood vessels form from preexisting blood vessels or de novo respectively. One technique involves the delivery of growth factors to stimulate endothelial cell recruitment^5–7^. However, these approaches are often limited by rapid diffusion, short half-lives, and poor biostability of growth factors^8^. An alternative tissue engineering approach involves the delivery of cells to directly differentiate into capillary structures. Various cell types have been shown to create new capillary networks *in vivo*^9–11^. In addition, some strategies involve implanting engineered scaffolds or co-delivering endothelial cells (ECs) with stromal cells to promote vessel in-growth or stable, mature vasculature formation, respectively^12–15^. Despite these advances, there are still critical challenges that plague their application, such as possible immunorejection from the host and the vast number of cells required for human translation^16–18^.

Advances in cellular reprograming have led to the discovery of one particularly exciting alternative cell source for therapeutic vascularization, induced pluripotent stem cells (iPSC). These cells are derived from reprograming adult somatic cells into pluripotency, a stem-cell like state, typically with four transcription factors [Oct4, Sox2, Klf4, and cMyc (OSKM)]^19^. In this state, cells can be differentiated into many different lineages, including the mesoderm to create endothelial cells^20^. iPSCs offer numerous advantages including their potential autologous nature, which could eliminate any immunological concerns during their therapeutic delivery. Furthermore, since these cells are derived from adult somatic cells, there is little ethical concern over their use, despite their stem cell-like lineage. Most importantly, these cells can be created from various sources and have an unlimited proliferation capacity, in theory, leading to a potentially large reservoir of cells for clinical applications^21^.

Research has successfully demonstrated the ability to differentiate iPSCs into endothelial cells^22–24^. These induced pluripotent stem cell-derived endothelial cells (iPSC-ECs) are characterized by their ability to express endothelial cell markers. Further studies with iPSC-ECs revealed their potential to form vessel-like networks on a Matrigel supporting material both *in vitro* and *in vivo*^25–27^. While this research is promising for tissue engineering and revascularization, very little is known about how these cells behave and compare to other endothelial cell sources, specifically in the quantity, quality, and function of the vessel-like networks formed.

The present study explores whether a potentially autologous EC source derived from human induced pluripotent stem cells (iPSC-ECs) can form the same robust, stable microvasculature previously documented for other sources of ECs. Using a well-established *in vitro* model, endothelial cells were coated on dextran microcarrier beads and co-embedded in a 3D fibrin matrix with normal human lung fibroblasts (NHLF). Fibrin was selected due to its naturally occurring presence in humans and FDA clearance for clinical use^28,29^, while NHLF were chosen due to their aforementioned ability to aid in the formation of microvascular networks as previously reported^30–32^. We examined differences in capillary morphogenesis of iPSC-ECs and human umbilical vein endothelial cells (HUVECs) by quantifying total network lengths, number of branch points, and number of vessel-like segments, and qualitatively identifying characteristics of mature capillaries. Functional and mechanistic differences were identified by mechanically or chemically inhibiting capillary morphogenesis with elevated fibrin concentrations or proteolytic inhibitors respectively. We also investigated key differences in matrix proteolysis, and identified a potentially significant mechanistic difference between iPSC-ECs and HUVECs that may influence the translational potential of the former.

## Results

### iPSC-ECs exhibit deficiencies in capillary morphogenesis compared to HUVECs

**Figure 1:**
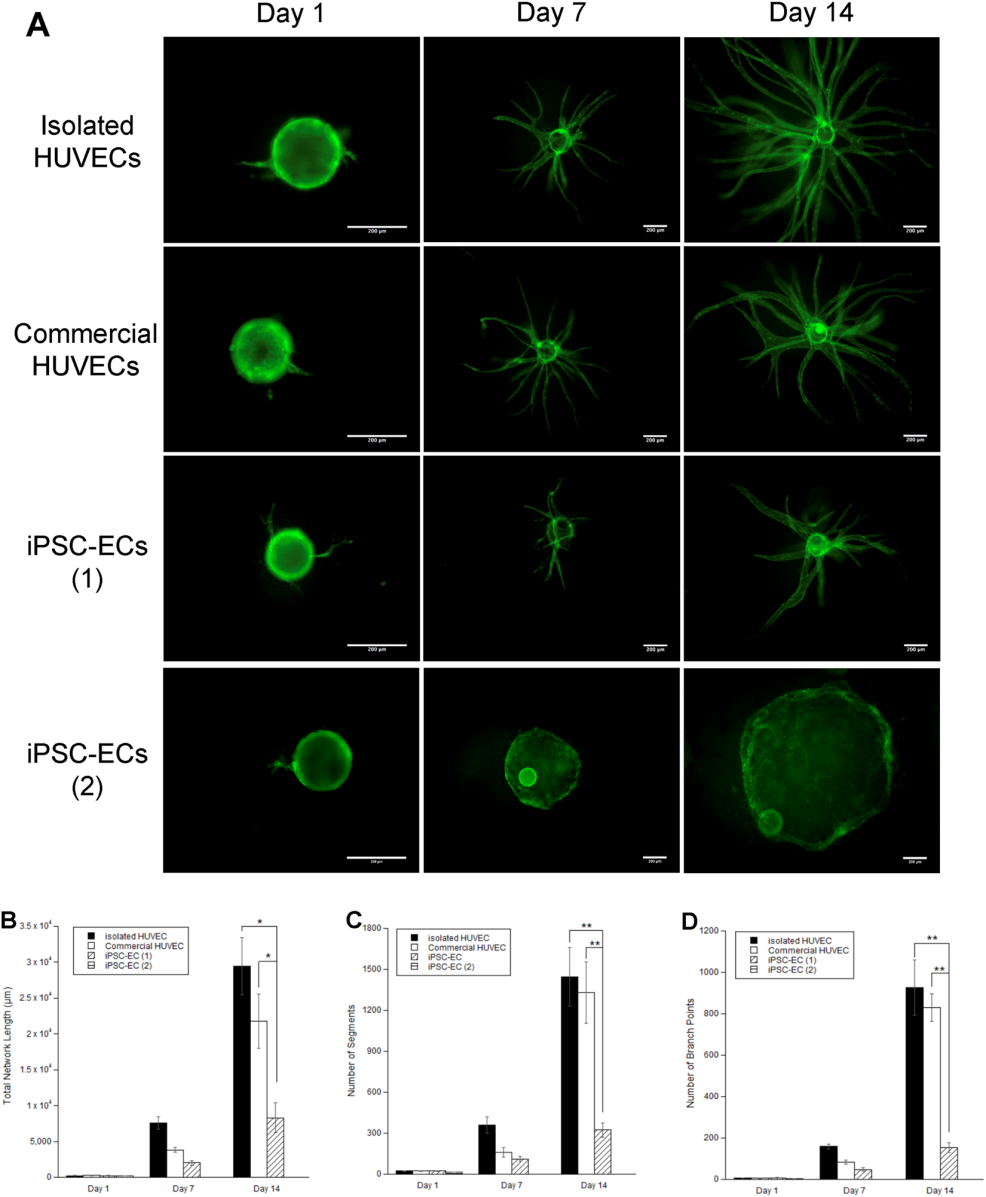
iPSC-ECs exhibit deficiencies in capillary morphogenesis compared to HUVECs. **(A)** EC-coated microbeads embedded in 2.5 mg/mL fibrin with NHLF at various time points were stained for CD31 and visualized via fluorescent microscopy. Scale bar = 200 µm. Over 3 separate experiments, a total of 30 beads per EC were quantified and averaged for **(B)** total capillary network length, **(C)** number of segments, and **(D)** number of branch points. *p < 0.05 and **p < 0.01 when comparing the indicated condition to the isolated HUVEC control at that time point. Error bars indicate ±SEM.

In-house isolated HUVECs (“isolated HUVECs”), commercially purchased HUVECs (“commercial HUVECs”), and two different sources of iPSC-derived ECs [iPSC-ECs (1) and iPSC-ECs (2)] were characterized for the ability to sprout from microcarrier beads when co-cultured with NHLFs in a 3D fibrin matrix. Immunofluorescent staining for CD31 in these cultures demonstrated successful attachment, invasion into the ECM, and primitive sprouting across all EC types at day 1. However, on days 7 and 14, the capillary sprouting of iPSC-ECs (1) showed significant reductions in their networks compared to the two HUVEC conditions (Fig. 1A), while no capillary sprouting was evident for iPSC-ECs (2). Quantification of these networks (Fig. 1B) demonstrated a significant decrease in total network length between the two HUVEC conditions and iPSC-EC (1) (8311 ± 2091 µm for iPSC-EC versus 29458 ± 3977 µm for the isolated HUVEC and 21794 ± 3825 µm for the commercial HUVECs on day 14). This reduced total network length was accompanied by a five-fold decrease in the number of vessel branch points and number of segments formed (Fig. 1C,D). iPSC-ECs (2) was not quantified at day 7 and day 14 time points due to the lack of sprouting. There was no statistical difference between the two HUVEC conditions, despite slightly reduced total network length, number of branch points, and number of segments. Since there were no statistical differences, subsequent experiments used only in-house isolated HUVECs, and henceforth are referred to as HUVEC solely. Furthermore, due to their ability to form at least some capillary-like networks, iPSC-ECs (1) was used as the sole iPSC-EC source in subsequent experiments and henceforth are referred to as iPSC-EC.

### iPSC-EC’s vessel-like structures express characteristics of mature capillaries

**Figure 2:**
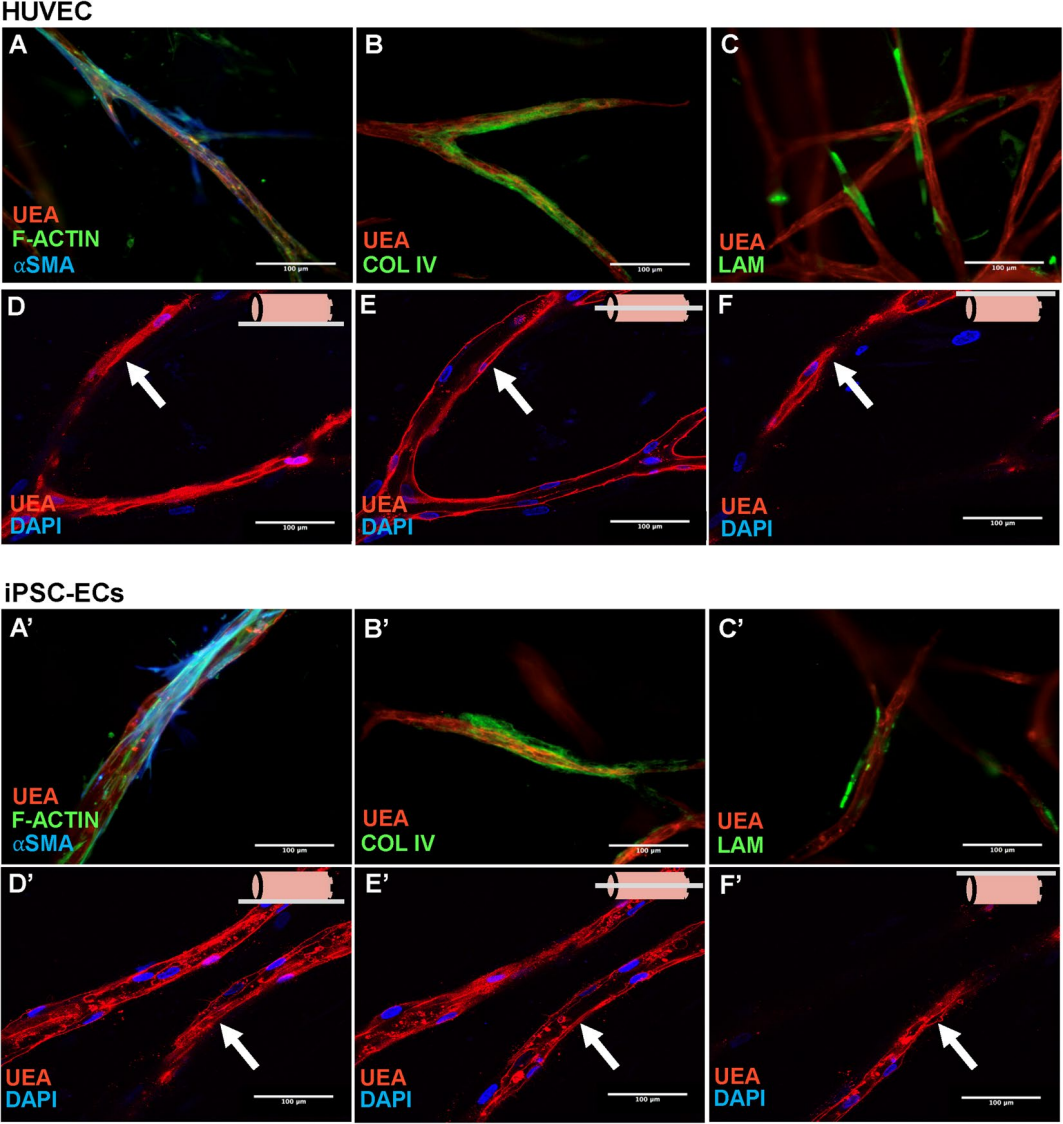
Both HUVECs and iPSC-ECs form vessel-like structures with characteristics of mature capillaries. HUVECs **(A–F)** or iPSC-ECs **(A’–F’)** were coated on micro carrier beads and embedded in a fibrin ECM with NHLFs interspersed throughout. Beads were monitored over a 14-day period. **(A**,**A’)**. Cultures were fixed and IF stained at day 14 for UEA (red), F-Actin (green), and αSMA (blue). Pericytic association was observed for both EC types. Cultures were fixed and IF stained at day 14 for **(B**,**B’)** UEA (red), and collagen IV (green) or **(C**,**C’)** UEA (red), and laminin (green). Basement membrane deposition was observed for both EC types. Hollow lumen formation was demonstrated through laser confocal microscopy at the bottom **(D**,**D’)**, middle **(E**,**E’)**, and top **(F**,**F’)** slice of vessel-like structures. The schematic in the upper right of each of these subsets indicates the slice relative to the vessel. Arrows indicate areas of focus. Scale bars = 100 µm.

Despite iPSC-ECs forming vessel-like structures, the quality of the structures was also examined to determine if they exhibit qualitative characteristics of mature capillaries. In 3D fibrin cultures, vessels of both endothelial cell types (HUVECs and iPSC-ECs), stained with UEA, were surrounded by basement membrane sleeves, as gauged by immunofluorescent staining for collagen IV (Fig. 2A and A’) and laminin (Fig. 2B and B’). Both collagen IV and laminin are prominent components in basement membranes^33^. Pericytes stabilize nascent endothelium and are characterized by physical association with ECs as well as expression of molecular markers such as αSMA^34^. IF staining revealed NHLFs associated with vessels formed from both HUVECs (Fig. 2C) and iPSC-ECs (Fig. 2C’) were positive for αSMA, suggestive of a pericyte-like phenotype when co-cultured within the fibrin matrix. Confocal analysis through multiple parallel focal planes of UEA-stained iPSC-EC and HUVEC cultures was used to verify the formation of hollow lumens. UEA staining was observed in a planar fashion in the bottom (Fig. 2D and D’) and top slices (Fig. 2F and F’), but only present on the borders in the middle slice (Fig. 2E and E’), indicative of EC differentiation into lumen-containing structures. Collectively, these results demonstrate that iPSC-ECs form vessel-like networks exhibiting characteristics of mature capillaries, similar to those formed by HUVECs in 3D co-cultures.

### Distributing stromal cells throughout matrix abrogates sprouting decreases for iPSC-EC capillary network formation in elevated fibrin concentrations

**Figure 3:**
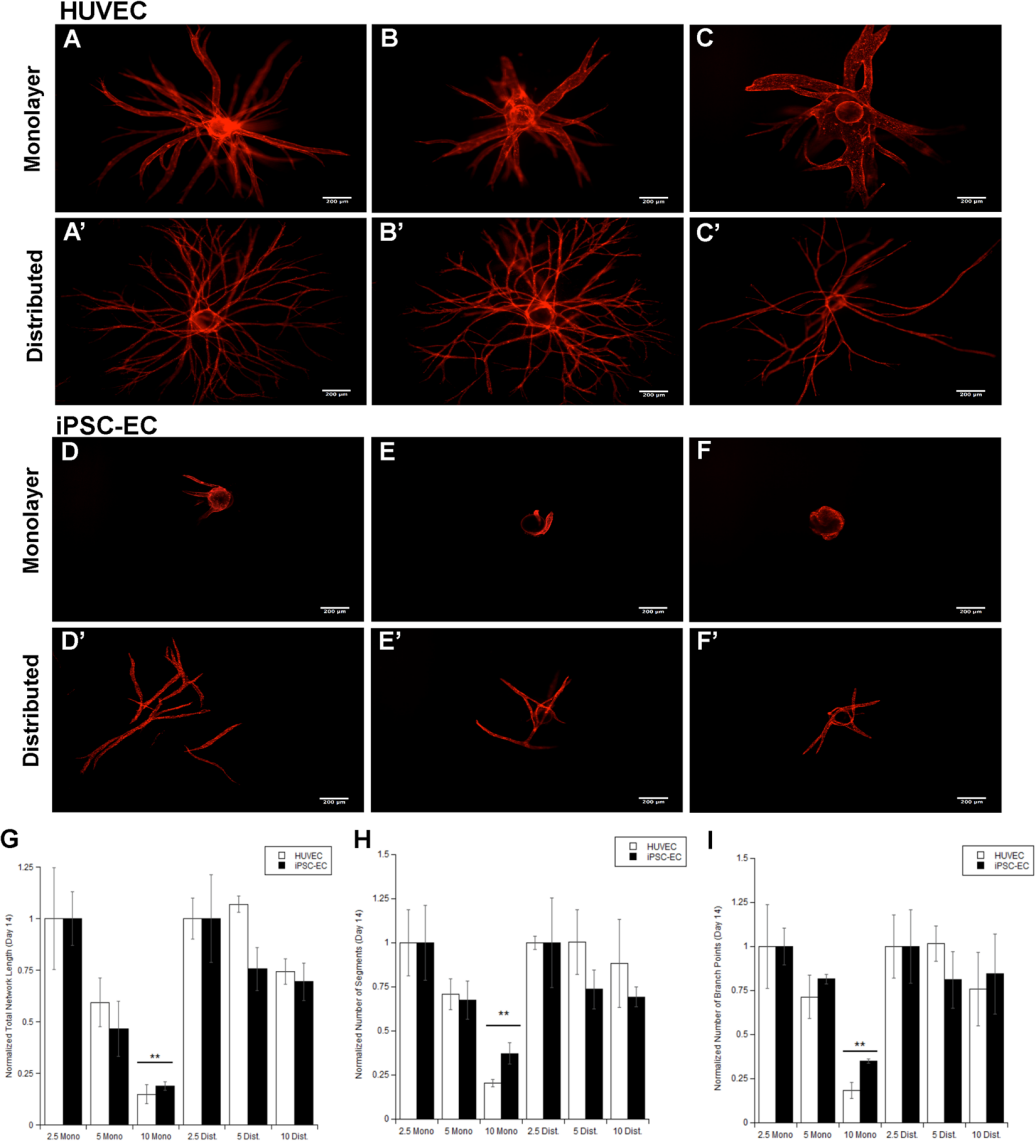
Distributing stromal cells throughout the matrix abrogates reductions in EC sprouting caused by elevated fibrin concentrations for both HUVECs and iPSC-ECs. Fluorescent images of UEA-stained HUVEC (**A**–**C**,**A**–**C**’) or iPSC-EC (**D**–**F**,**D**’–**F**’) coated microcarrier beads with (**A**–**F**) overlaying monolayer or (**A**’–**F**’) distributed NHLFs. Beads are embedded in (**A**,**A**’,**D**,**D**’) 2.5 mg/mL, (**B**,**B**’,**E**,**E**’) 5 mg/mL, (**C**,**C**’,**F**,**F**’) 10 mg/mL fibrin matrices. Scale bar = 200 µm. A total of 30 beads over three separate experiments at day 14 were quantified, averaged, and normalized to the respective 2.5 mg/mL stromal cell distribution of each EC type for (**G**) total capillary network length, (**H**) number of segments, and (**I**) number of branch points. *p < 0.05 and **p < 0.01 when comparing the indicated condition to the 2.5 mg/mL monolayer condition. Error bars indicate ±SEM.

Previous research has shown that elevated fibrin concentrations have an inhibitory effect on HUVEC capillary morphogenesis^30^. To investigate any functional similarities between iPSC-EC vasculature, ECs were cultured on microcarrier beads within 2.5 mg/mL, 5 mg/mL, and 10 mg/ml fibrin gels overlaid with a NHLF monolayer. Extensive capillary networks stained for UEA were formed from both HUVECs (Fig. 3A) and iPSC-ECs (Fig. 3D) by day 14. Increasing matrix density to 5 mg/mL inhibited network formation slightly for both ECs (Fig. 3B and E), while greatly inhibiting sprouting for both ECs in 10 mg/mL matrices (Fig. 3C and F). Quantification of these networks, normalized to their respective 2.5 mg/mL condition, demonstrated a significant decrease in total network length (Fig. 3G), number of vessel segments (Fig. 3H), and number of vessel branch points (Fig. 3I) between both ECs 2.5 mg/mL conditions and their respective 10 mg/mL condition. Research suggests that for HUVECs, distributing the fibroblasts throughout the matrix abrogated the inhibitory effect of increased fibrin concentration, primarily by overcoming diffusion limitations imposed on ECs in denser matrices with stromal cell monolayers^30^. Thus, to assess whether the same effect is seen with iPSC-EC capillary morphogenesis, we distributed fibroblasts throughout the matrix embedded with EC-coated microcarrier beads. IF staining for UEA revealed a notable increase in network formation for both EC types across all matrix densities (Fig. 3A’–F’). Furthermore, quantification of total network length (Fig. 3G), number of vessel segments (Fig. 3H), and number of vessel branch points (Fig. 3I), normalized to their respective 2.5 mg/mL condition, no longer decreased significantly with increasing fibrin densities. Collectively, the inhibition of morphogenesis through elevated matrix density, and the subsequent abrogation of inhibition by direct co-culture of ECs with stromal fibroblasts, demonstrates that iPSC-EC vascular networks are functionally regulated in a manner similar to HUVEC networks.

### Capillary morphogenesis by iPSC-ECs involves both plasmin-mediated and MMP-mediated mechanisms

**Figure 4:**
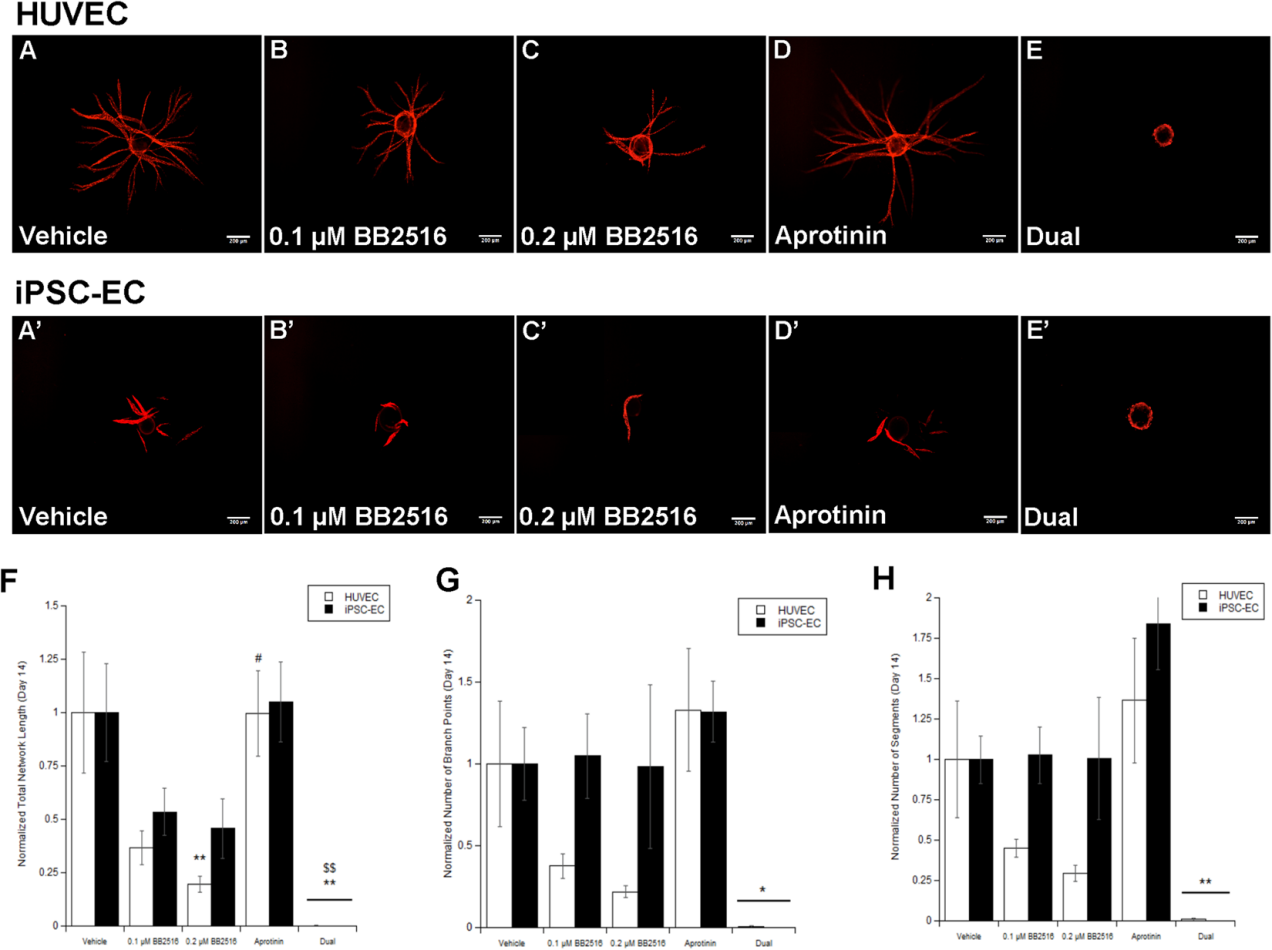
Capillary morphogenesis by iPSC-ECs and HUVECs proceed via similar preoteolytic mechanisms. HUVEC **(A–E)** or iPSC-EC **(A’–E’)** coated microcarrier beads were embedded in a fibrin matrix dispersed with NHLFs. Shown are fluorescent images stained for UEA at day 14 of the capillary network formation from cultures treated with **(A**,**A’)** vehicle (DMSO), **(B**,**B’)** 0.1 µM, or **(C**,**C’)** 0.2 µM of the broad spectrum MMP inhibitor BB2516, **(D**,**D)** 22 nM of the serine protease inhibitor aprotinin, or with a combination of BB2516 (0.1 µM) and aprotinin (22 nM) (“dual”). Scale = 200 µm. **(F)** Total capillary network length, **(G)** number of segments, and **(H)** number of branch points from a minimum of 30 beads over three separate experiments at day 14 were quantified, averaged, and normalized to the respective EC vehicle control. *p < 0.05 and **p < 0.01 when comparing the indicated condition to the vehicle control. ^@^p < 0.05 and ^@@^p < 0.01 when comparing the indicated condition to the 0.1 µM BB2516 condition. ^#^p < 0.05 and ^##^p < 0.01 when comparing the indicated condition to the 0.2 µM BB2516 condition. ^$^p < 0.05 and ^$$^p < 0.01 when comparing the indicated condition to the aprotinin condition. Error bars indicate ±SEM.

Proteases play a key role in degrading and remodeling the ECM in capillary morphogenesis^35–37^. Our prior findings demonstrate capillary morphogenesis of HUVEC-NHLF co-cultures in fibrin gels proceeds in manner that involves both MMP- and plasmin-mediated fibrinolysis^38^. To assess the involvement of these proteases in iPSC-EC capillary morphogenesis, chemical inhibitors dissolved in a vehicle (DMSO) were added to cultures of 2.5 mg/mL fibrin gels embedded with EC-coated microcarrier beads and distributed fibroblasts. Gels were fixed, stained for UEA, and imaged at day 14 for both HUVECs (Fig. 4A–E) and iPSC-ECs (Fig. 4A’–E’). The addition of a broad MMP inhibitor, BB2516, at concentrations of 0.1 µM and 0.2 µM, did not significantly reduce sprouting in iPSC-EC cultures compared to the vehicle control (Fig. 4F). The resulting stasis in network formation is primarily attributed to no significant change in the number of branch points (Fig. 4G) and number of segments (Fig. 4H). While spouting was significantly reduced with 0.2 µM of BB2516 in HUVEC cultures (Fig. 4F), spouting was not completely eliminated, which is consistent with our previous findings^38^. For both HUVECs and iPSC-ECs, the serine protease inhibitor aprotinin, also did not alter network formation. However, the dual application of BB2516 (0.1 µM) and aprotinin (22 nM) completely eliminated network formation (Fig. 4E,E’,F), branching (Fig. 4G), and segmentation (Fig. 4H) in both EC types. In sum, these data demonstrate iPSC-ECs undergoing capillary morphogenesis stimulated by fibroblasts in 3D fibrin gels display similar proteolytic dependencies as HUVECs.

### iPSC-EC/NHLF co-cultures show differences in MMP RNA expression, protein expression, and activity levels compared to HUVEC/NHLF co-cultures

**Figure 5:**
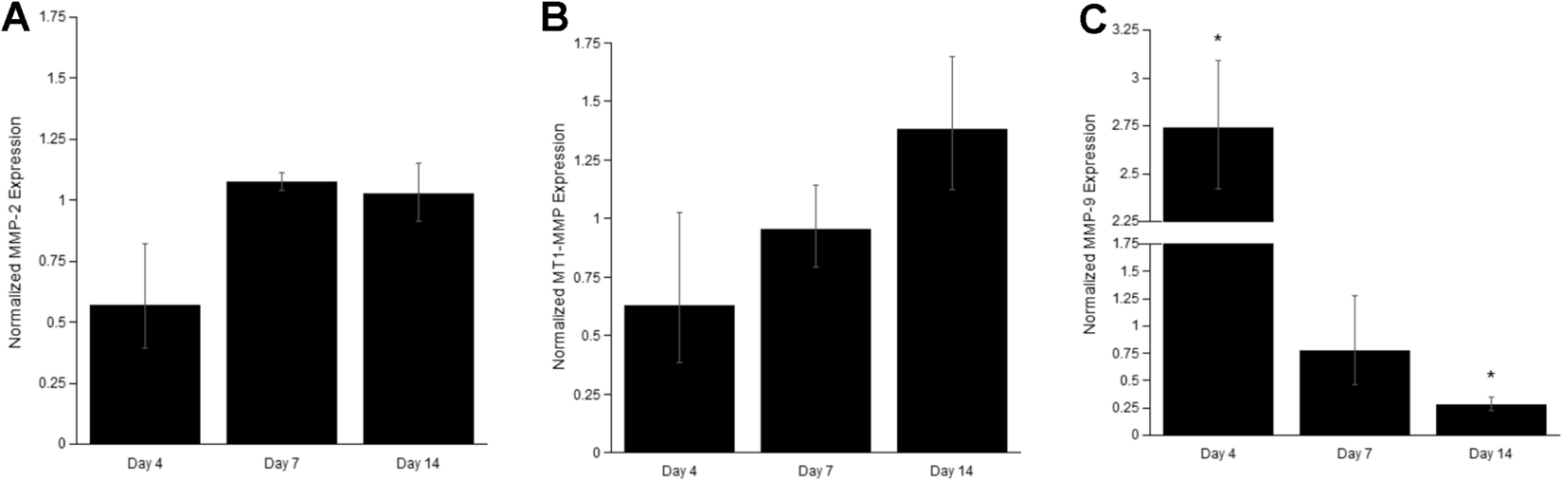
iPSC-ECs co-cultures show differences in MMP RNA expression levels compared to HUVEC co-cultures. The expression levels of key matrix metalloproteases [**(A)** MMP-2, **(B)** MT1-MMP, and **(C)** MMP-9] involved in capillary morphogenesis were quantified from iPSC-EC/NHLF co-cultures via qPCR. Expression levels were averaged across three separate experiments at the indicated time points and normalized to HUVEC/NHLF co-culture controls. *p < 0.05 when comparing the indicated time point to the HUVEC control. Error bars indicate ±SEM.

The expression of MMPs, specifically MMP-2, MMP-9, and MT1-MMP, in HUVECs is directly related to the formation of vessel-like networks^35,38,39^. Knockdown of MT1-MMP expression in particular results in attenuation of sprouting^35^. Despite similar dependencies on MMP- and plasmin-mediated mechanisms revealed through the use of protease inhibitors, we sought to compare the expression levels of MMPs between iPSC-EC and HUVEC co-cultures as a potential explanation for the attenuated sprouting in the case of the former. qPCR demonstrated no significant differences in the RNA expression levels of MMP-2 (Fig. 5A) and MT1-MMP (Fig. 5B) across all time points. However, MMP-9 levels (Fig. 5C) were significantly different in iPSC-EC co-cultures. At earlier times points (day 4), iPSC-EC co-cultures show a ~3.5 fold increase in RNA expression, while at later time points (day 14), a ~4 fold reduction in RNA expression.

**Figure 6:**
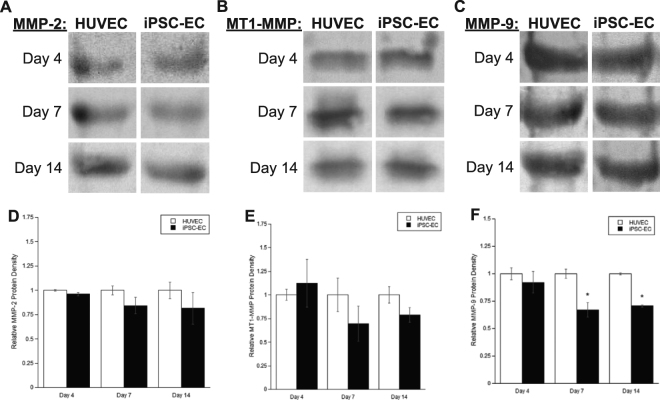
iPSC-EC/NHLF co-cultures show differences in MMP protein expression levels compared to HUVEC/NHLF co-cultures. Representative images of Western blots for **(A)** MMP-2, **(B)** MT1-MMP, **(C)** MMP-9 from HUVEC or iPSC-EC coated microcarrier beads co-cultured with NHLFS at various time points. Images were quantified and averaged across three separate experiments via scanning densitometry. Protein levels for **(D)** MMP-2, **(E)** MT1-MMP, and **(F)** MMP-9 were normalized to their respective HUVEC co-culture controls. *p < 0.05 when comparing the indicated time point to the HUVEC control. Error bars indicate ±SEM. Full (uncropped) Western blot images are shown in supplemental information (Fig. S1).

We next characterized MMP protein expression levels with Western blotting. MMP-2 (Fig. 6A), MT1-MMP (Fig. 6B), and MMP-9 (Fig. 6C) were all expressed at the protein level in iPSC-EC co-cultures. Semi-quantification of the band intensities revealed no significant differences in protein expression for MMP-2 (Fig. 6D), MT1-MMP (Fig. 6E), and at day 4 for MMP-9 (Fig. 6F). However, at later time points (day 7 and day 14), the protein expression levels of MMP-9 were significantly reduced in iPSC-EC co-cultures (~25% in both conditions), consistent with the qPCR results.

**Figure 7:**
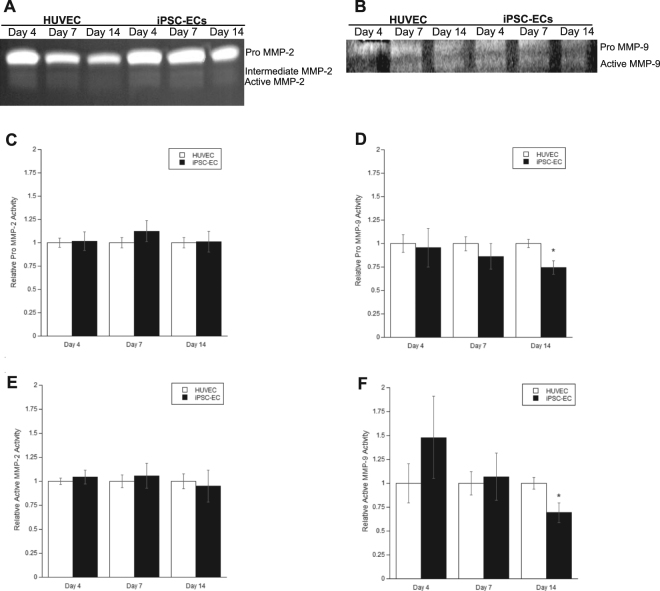
iPSC-EC/NHLF co-cultures show differences in the levels of MMP activity compared to HUVEC/NHLF co-cultures. HUVEC or iPSC-EC coated microcarrier beads co-cultured with NHLFs were digested and pooled to assay for activity via gelatin zymography. Representative images of zymograms performed at various time points for **(A)** MMP-2, and **(B)** MMP-9. A standard for MMP-2 and -9 was used to identify bands for pro-MMP-9 (92 kDa), active MMP-9 (88 kDa), pro-MMP-2 (72 kDa), intermediate MMP-2 (64 kDa), and active MMP-2 (62 kDa). Images were quantified, and averaged across three separate experiments via scanning densitometry. The levels for **(C)** pro-MMP-2, **(D)** pro-MMP-9, **(E)** active-MMP-2, and **(F)** active-MMP-9 were normalized to their respective HUVEC/NHLF co-culture controls. *p < 0.05 when comparing the indicated condition to the HUVEC control. Error bars indicate ±SEM. Images were set to 8-bit color and contrast enhanced in an identical manner for each gel prior to quantification. Representative enhanced images are shown here. Full unedited gelatin images are shown in supplemental information (Fig. S2).

Finally, the proteolytic activities of the MMPs in question were investigated using gelatin zymography. MMP-2 (Fig. 7A) and MMP-9 (Fig. 7B) both degraded a gelatin matrix across all time points in iPSC-EC co-cultures and HUVEC co-cultures. Semi-quantification via densitometry indicated no discernible differences in both pro- (Fig. 7C) and active- (Fig. 7E) forms of MMP-2. While there were no significant differences in pro- (Fig. 7D) and active- (Fig. 7F) forms of MMP-9 at earlier time points (day 4 and day 7), both forms of MMP-9 were significantly less active at day 14 in iPSC-EC co-cultures. Collectively, this data not only demonstrates the expression and activity of MMP-9 is significantly different in iPSC-EC co-cultures, but offers one potential mechanism to explain the attenuated capillary morphogenesis by iPSC-ECs.

## Discussion

A number of different sources of endothelial cells have been explored for their ability to revascularize ischemic wounds or build microvasculature in engineered tissues. HUVECs are a robust source of ECs with proven capability of capillary morphogenesis, particularly in the assay used here, but these cells are not without their limitations. Their venous origins are often cited as a potential problem, despite evidence in the literature that ECs in the arterial circulation arise from a venous origin in development^40^. Furthermore, HUVECs and human microvascular ECs (HMVECs) have exhibited similar revascularization capacities in several studies^41,42^. Two potentially more critical limitations of HUVECs (and also HMVECs) are their allogeneic origin and their limited proliferation potential, especially given the need to generate the large numbers of cells, on the order of billions^43,44^, for human applications.

As a consequence of the perceived limitations of existing EC populations, the vascularization potential of iPSC-ECs as a more clinically relevant cell source is increasingly of interest. Prior studies have shown that iPSC-ECs are capable of forming vessel-like structures both *in vitro* and *in vivo* within supporting Matrigel matrices^25–27^, but there is little (if any) evidence comparing their potential side-by-side with more widely utilized EC sources. This study therefore explored the ability of iPSC-ECs to create functional vessel-like structures in a clinically relevant 3D *in vitro* model of angiogenesis^30,35,38,39,45–47^. We have shown that iPSC-ECs coated on microcarrier beads embedded in fibrin with NHLFs yield networks with significantly shorter total network lengths (a quantitative measure of the extent of capillary morphogenesis) compared to HUVECs. If iPSC-ECs cannot efficiently yield microvascular networks of sufficient quantity, diseased or necrotic tissue may not be effectively revascularized in a timely manner, suggesting significant scientific barriers must be overcome in order for the translational potential of these cells to be realized.

In addition to the side-by-side comparisons of iPSC-ECs and HUVECs in this study, we also examined two different sources of both iPSC-ECs and HUVECs. While both iPSC-EC sources resulted in reduced capillary network formation, the variation in sprouting between the two populations demonstrates the need for standardization of iPSC-EC production. Once again, the clinical potential for iPSC-ECs is promising and could revolutionize the field of therapeutic angiogenesis. However, without a standardized approach to differentiate iPSC into an EC lineage, the success of clinical translation may vary significantly, just as the angiogenic sprouting we observed here varied significantly with different iPSC-EC sources. As for the HUVECs, we observed only slight differences between the two sources, especially on day 7, which may be attributable to the homogeneity of the source. Our in-house isolated HUVECs were isolated from a single umbilical cord while the commercial source was a population of cells pooled from multiple cords. Furthermore, the commercial HUVECs were pooled from donors of more than one gender, and there are reported differences in HUVECs isolated from different sexes^48^. The in-house isolated HUVECs, from a single source, represent a more appropriate comparison to the iPSC-ECs given the expectation that iPSC-ECs might be derived from a patient, i.e. a single autologous cell source.

While iPSC-ECs exhibit quantitative deficiencies in sprouting, the vessel-like structures formed displayed characteristics of mature capillaries. Collagen IV and laminin are components of the basement membrane of mature capillaries^33^, and thus the presence of these components is indicative of iPSC-ECs’ ability to form mature capillary networks. Collagen IV was observed surrounding the nascent vessels completely, while the laminin coverage was more sparse. The spatial distribution may be an artifact of confocal imaging, as multiple z-plane images were flattened on top of one another to create a single image; nevertheless the presence of both collagen IV and laminin suggest the iPSC-ECs can deposit a basement membrane. In addition, the NHLFs associate with the vessel-like structures formed from iPSC-ECs in a pericyte-like manner, which may serve to stabilize nascent vasculature^34,38^. Importantly, this data suggest iPSC-ECs can recruit and signal stromal cells to differentiate into pericytes. Furthermore, the presence of hollow lumens in the iPSC-EC capillary-like networks demonstrates their potential to be perfused and, once again, suggests iPSC-ECs may be capable of attaining a more mature phenotype.

The inhibition, and subsequent abrogation of inhibition, of iPSC-EC capillary morphogenesis in fibrin gels of elevated concentrations demonstrates the functional similarities of the vessel-like structure formed to their HUVEC counterparts. In ECMs of higher density, transport from stromal fibroblasts to ECs is inhibited, which significantly affects capillary morphogenesis when the fibroblasts are cultured on top of the gels at a fixed distance away from the ECs^34^. Distributing the stromal cells throughout the gel reduces these transport limitations, allowing for greater network formation. The data were normalized to their respective 2.5 mg/mL condition to highlight the similarities between iPSC-ECs and HUVECs, since the iPSC-EC condition was otherwise dwarfed in magnitude by the HUVEC condition on a non-normalized scale. Qualitatively, we also observed wider vessels formed with increased fibrin concentrations. This may be attributable to the reduced porosity of the matrix increased densities being less porous^45^. Decreased pore size may cause cells to proliferate radially instead of being able to expand throughout the matrix. Another possibility is that the ligand binding density has morphogenetic effects on these cells, which are abrogated by signaling from the stromal cells^49^. Regardless, the effects of fibrin concentration and stromal cell distribution on network formation demonstrate the phenotypic similarities between the two EC populations.

Broad spectrum protease inhibition revealed iPSC-EC/NHLF co-cultures utilize similar proteolytic mechanisms. The fibroblast-mediated sprouting was only completely attenuated through dual inhibition of both MMPs and serine proteases, which is consistent with findings we previously reported for HUVEC/NHLF co-cultures^38^. This proteolytic plasticity could be explained by the relationship between plasmin and MMPs^50^ or possibly a third type of extracellular protease involved in the morphogenic process^51^. Of the two doses of MMP inhibitor (BB2516) we tested, the higher dose (0.2 µM) did significantly reduce total network lengths in the HUVEC/NHLF co-cultures compared to vehicle controls, consistent with our prior findings^38^, but did not significantly affect the iPSC-EC/NHLF co-cultures. Of course, the overall sprouting of the latter cultures is significantly less than the former, and thus any subtle effects of the protease inhibitors may be more difficult to detect.

The experiments with elevated fibrin concentration and the protease inhibitors suggested that iPSC-ECs and HUVECs utilize the same fibrinolytic mechanisms. However, we hypothesized that differential quantities and/or activities of key MMPs may underlie the attenuated iPSC-ECs sprouting relative to HUVECs. qPCR showed the expression levels of mRNAs encoding for MMP-2 and MT1-MMP were similar between HUVEC and iPSC-EC co-cultures, and did not vary significantly with time. By comparison, MMP-9 mRNA levels were significantly higher in iPSC-EC than HUVEC co-cultures initially, but then dropped off dramatically over the time course of the sprouting assay to become significantly lower than the HUVEC by day 14. These findings were mirrored in terms of protein levels and enzymatic activities, with the levels and activities of MMP-9 significantly less in iPSC-EC co-cultures by day 14 relative to HUVECs. While there is compelling evidence that the MT-MMPs may be the only essential MMPs for capillary morphogenesis^37^, multiple MMP family members, including MMP-9, have been implicated in capillary invasion in fibrin gels^52^ and there is evidence that MT1-MMP regulates MMP-9 specifically^53^. Therefore, it is entirely plausible the reduced MMP-9 expression and/or activity contribute to the inability of the iPSC-ECs to form capillary-like networks of the same magnitude as HUVECs. While molecular genetics tools to knock-down and/or over-express MMP-9 in iPSC-EC may offer a more definitive interpretation of our findings here, the key focus of this study was to compare iPSC-ECs head-to-head with an established EC source.

One caveat to interpreting the observed differences in iPSC-ECs’ vasculogenic potential is the fact only one source of iPSC-ECs was capable of capillary morphogenesis. While multiple lots and sources were tested and showed reduced network formation, it is possible that variations in proprietary differentiation techniques could influence the potential of these cells. On the other hand, a notable difference between HUVECs and iPSC-ECs is the fact the former have been exposed to blood flow, while the latter have not. Given the role of shear stresses on vascular development^54^, it seems reasonable the iPSC-ECs represent an immature EC phenotype that may require mechanobiological cues to fully differentiate into ECs capable of robust branching morphogenesis in response to angiogenic stimuli. If indeed this is true, then clearly these cues (or their absence) should be considered when interpreting data involving the use of iPSC-ECs for drug discovery and toxicity testing. It is also important to note our assessment of differences in the repertoire of proteolytic enzymes was performed in EC/NHLF co-cultures. While it is possible, though technically challenging, to separate the relative contributions of these two populations of cells, we elected not to do so given the importance of reciprocal cross-talk between ECs and their stromal support cells^55^ and our intention to co-deliver both cell types *in vivo* for revascularization applications. Finally, while we focused on proteolytic differences between iPSC-ECs and HUVECs to partially explain the attenuation of sprouting, we of course cannot rule out other possibilities.

A prior study from our group has shown that stromal cells of different origins induce ECs to form more mature capillaries characterized by less extravascular leakage, the expression of mature pericyte markers, and more tightly regulated permeability^56^. Stromal cells other than NHLFs may therefore be better able to induce iPSC-EC capillary morphogenesis. Similarly, it may be possible to enhance the angiogenic potential of iPSC-ECs using matrix materials other than fibrin. Synthetic hydrogels have been shown to support iPSC-EC capillary morphogenesis already^57,58^, and controlling their mechanical properties may represent a means to increase the expression of vasculogenic and proteolytic genes as recently reported for ECs derived from human embryonic stem cells^59^. Collectively, these possibilities suggest key features of the microenvironment may be manipulated to enhance the therapeutic potential of the iPSC-ECs.

In summary, this work assessed whether iPSC-ECs form the same robust, stable microvasculature as previously documented for other sources of EC in a well-characterized 3D fibrin-based co-culture model of angiogenic sprouting *in vitro*. Both HUVECs and iPSC-ECs formed vessel-like networks with some characteristics of mature microvasculature, and utilized similar proteolytic invasion mechanisms. However, significant attenuation of sprouting by iPSC-ECs (vs. HUVECs) was observed, and we identified differences in the expression levels of MMP-9 as a possible mechanistic explanation. Future *in vivo* studies are necessary to determine if this attenuation is only an *in vitro* phenomenon. Ultimately, despite the promise and potential of iPSC-ECs for therapeutic revascularization, these findings suggest fundamental phenotypic differences must be understood to enable pre-clinical and clinical translation.

## Materials and Methods

### HUVEC Isolation and Cell Culture

Human umbilical vein endothelial cells were either purchased (Lonza, Walkersville, MD) or harvested from fresh umbilical cords from the University of Michigan Mott Children’s Hospital via an IRB-exempt protocol and isolated from methods previously described^45^. Briefly, the umbilical cord was rinsed in phosphate buffer saline (PBS) and then digested with 0.1% collagenase type I (195 U/ml, Worthington Biochemical, Lakewood, NJ) for 20 min at 37 °C. The digested product was subsequently washed in PBS, collected, and centrifuged (200 × G for 5 min). The pellet was resuspended in endothelial growth media (EGM-2, Lonza), and the cells were plated in tissue culture flasks and cultured at 37 °C and 5% CO_2_. After 24 hours, HUVECs were rinsed with PBS to remove any non-adherent cells. Fresh media was changed every 48 hours. Cells from passage 3 were utilized for experiments. Normal human lung fibroblasts (NHLF, Lonza) were cultured at 37 °C and 5% CO_2_ in Dulbecco’s modified eagle media (DMEM, Life Technologies, Grand Island, NY) with 10% fetal bovine serum (FBS). Culture media was replaced every 48 hours and cells from passage 6–10 were used in experiments. Two sources of iPSC-ECs were used in our experiments. iCell endothelial cells (Cellular Dynamics International, Madison, WI), referred to as iPSC-ECs (1), were cultured at 37 °C and 5% CO_2_ in Vasculife VEGF endothelial media (Lifeline Cell Technology, Fredrick, MD) supplemented with iCell Endothelial Cell Medium Supplement (Cellular Dynamics International). Three different lots were used, and all of the data generated with each lot of cells combined together. A second source of iPSC-ECs, referred to as iPSC-ECs (2), were graciously provided by Dr. Ngan Huang (Stanford University). iPSC-EC (2) were cultured at 37 °C and 5% CO_2_ in EGM-2MV (Lonza). Both iPSC-EC tissue culture flasks were coated with 35 µg/mL fibronectin (Invitrogen, Carlsbad, CA) for 1 hr at room temp prior to plating the cells. Culture media was replaced every 48 hours and cells from passage 3 were used in experiments.

### Microcarrier Bead Assembly

Cytodex microcarrier beads (Sigma-Aldrich, St. Louis, MO) were hydrated and sterilized in phosphate buffer saline (PBS). Beads were prepared for coating by washing repeatedly with 1 mL of EGM-2, with time to settle between washes. Endothelial cells were cultured in T-75 flasks to 80% confluency and rinsed with PBS before being harvested via 0.25% trypsin incubation for 5 min at 37 °C and 5% CO_2_. Trypsin was neutralized using DMEM supplemented with 10% FBS. The cellular suspension was centrifuged (200 × G for 5 min) and supernatant was aspirated immediately. The cell pellet was re-suspended in 4 mL of fresh EGM-2. 10,000 microcarrier beads were combined with four million ECs, HUVEC or iPSC-EC, (5 mL total) in an inverted T-25 culture flask. Over a 4 hour incubation period, the culture flask was agitated every 30 minutes to ensure EC coating of beads. After 4 hours, the cell-bead mixture was added to a new T-25 culture flask. Fresh EGM-2 (5 mL) was added to the old flask to remove any remaining beads and transferred to the new culture flask. The total volume (10 mL) was incubated overnight in standard cell culture position.

### Fibrin Tissue Assembly

The next day, following bead coating, a fibrinogen (Sigma-Aldrich) solution of the desired concentration (2.5 mg/mL, 5 mg/mL, or 10 mg/mL, based on desired experimental conditions) was dissolved in an appropriate amount of serum-free EGM-2 and placed at 37 °C in a water bath. The solution was sterile filtered through a 0.22 µm syringe filter (Millipore, Billerica, MA). The previous day’s cell-bead solution was removed from the culture flask and placed in a 15 mL centrifuge tube. After the beads settled, the remaining supernatant was used to remove any remaining beads adhering to the culture flask and added to the centrifuge tube. Upon the beads settling, the supernatant was removed and 5 mL of fresh serum-free EGM-2 was added to the cell-coated beads. The appropriate amount of bead solution (~50 beads per well) was added to the fibrinogen solution with 5% FBS. Fibroblasts were prepared using a similar rinsing/trypsinization procedure as described above. 25,000 NHLFs per well were added to the bead-fibrinogen solution or plated on top of each gel after polymerization in our distributed and monolayer conditions respectively. 500 µL of above mixture was added to a single well of a 24-well tissue culture plate and polymerized with 10 μL of thrombin (50 U/mL, Sigma-Aldrich). Tissue constructs were left undisturbed for 5 min at room temperature before incubation for 30 min at 37 °C and 5% CO2. For studies involving inhibitors, the appropriate vehicle or inhibitor(s) was mixed with the media prior to addition to the culture. 1 mL of fresh EGM-2 (±chemical inhibitors) was added on top of the gels following incubation and changed the following day and every other day thereafter. The media was changed to serum free EGM-2 two days prior to harvesting for protein and RNA analysis.

### Immunofluorescent staining

After the constructs were cultured for a specified period of time (1, 4, 7, or 14 days), gels were rinsed 3× with PBS solution for 5 min at room temperature. Gels were then fixed with 500 μL of formalin (1 mL of 36.5% Formaldehyde solution (Sigma), 1 mL of PBS, and 8 mL of d.d.H2O) for 15 min at 4 °C. Gels are rinsed again 3× with PBS for 5 min, then permeabilized with 0.5% Triton-X100 in TBS for 30 min at 4 °C. Following a rinse 3× for 5 min at room temperature with 0.1% Triton X-100 in TBS (TBS-T), samples were blocked overnight at 4 °C with a 2% Abdil solution (bovine serum albumin (Sigma) dissolved in TBS-T). The primary antibody/staining agent was dissolved in 2% Abdil at the appropriate concentration (Ulex Europaeus Lectin 1 (UEA), 1:100 (Vector Labs, Burlingame, CA); anti-CD31, 1:200 (Dako, Santa Clara, CA); collagen IV, 1:200 (Pierce Biotechnology, Waltham, MA); laminin, 1:200 (Pierce Biotechnology); alpha-smooth muscle actin (αSMA), 1:200 (Pierce Biotechnology)) and 1 mL of this solution was added to each gel for overnight incubation at 4 °C. The following day gels were rinsed 3× for 5 min with TBS-T. 1 mL of the appropriate secondary antibody (1:400, Alexa Fluor 488 Goat anti-mouse IgG, Alexa Fluor 405 Goat anti-mouse IgG, Alexa Fluor 488 Goat anti-rabbit IgG, Alexa Fluor 405 Goat anti-rabbit IgG, Invitrogen) dissolved in 2% Abdil was added to each gel for overnight incubation at 4 °C. Following a 3× rinse for 5 min at room temperature with TBS-T, gels are incubated with TBS-T overnight at 4 °C.

### Fluorescent Imaging and Vessel Quantification

Vessel formation was assessed at the aforementioned time points. Fluorescent images were captured utilizing an Olympus IX81 equipped with Disc Spinning Unit and a 100 W high-pressure mercury burner (Olympus America, Center Valley, PA), a Hamamatsu Orca II CCD camera (Hamamatsu Photonics, K.K., Hamamatsu City, Japan), and Metamorph Premier software (Molecular Devices, Sunnyvale, CA). Imaged beads were chosen at random provided that vessels emanating from a given bead did not form anastomoses with vessels from adjacent beads. Images from at least 30 beads per condition were captured over three separate trials at low magnification (4×) for each independent experiment and processed using the Angiogenesis Tube Formation module in Metamorph Premier (Molecular Devices). Each image was segmented and analyzed based on any tube-like pattern that falls within a specified minimum and maximum width of each segment above a contrast threshold. The total network length, the number of branch points, and number of segments were quantified.

### Fibrin Gel Lysing

For each EC type, the spent media was collected from ten fibrin gels, each containing ~100 EC-coated beads. Each gel was then washed with PBS. The gels were then dislodged from each well in the plates using a small spatula to allow for optimal dissolution of the gels. Fibrin gels were dissolved using 500 μL of Nattokinase (1000 U/mL, Japan Bio Science Laboratory Co., Osaka City, Japan) and incubated at 37 °C for 45 min with agitation periodically. To ensure complete removal of the ECs from the microcarrier beads, the contents of each well were pipetted repeatedly. The solution was removed and centrifuged at 200 × G for 5 min to collect the ECs. Supernatant was aspirated and cells were lysed and suspended in RIPA lysis buffer (50 mM Tris-HCl pH 7.6, 150 mM NaCl, 1% Triton X-100, 0.5% sodium deoxycholate, 0.1% SDS) before storage at −80 °C. Bicinchoninic acid assay (Pierce Biotechnology, Rockford, IL) was utilized to determine the protein concentration of the lysed cells and supernatant.

### Western Blotting Analysis

Western blot analysis of the levels of MT1-MMP was conducted on the lysed tissue samples, while the levels of MMP-2, and MMP-9 were assessed in the spent media. After boiling, equal amounts of protein (25 µg) from the respective samples were electrophoresed in a 10% Tris-glycine gel (Invitrogen) under reducing conditions and transferred to a PVDF membrane. Blots were probed in a 5% Abdil solution with mouse monoclonal antibodies for human MMP-2 (1:1000, Abcam, Cambridge, UK) and human MMP-9 (1:1000, Abcam) or rabbit monoclonal antibodies for human MT1-MMP (1:2000, Abcam). Blots were incubated for two hours at 25 °C with gentle agitation and subsequently washed 6× with TBS-T for 5 min. After washing, the membrane was incubated in TBT-T with horseradish peroxidase-conjugated anti-mouse secondary antibody (1:10,000, Pierce Biotechnology) or horseradish peroxidase-conjugated anti-rabbit secondary antibody (1:10,000, Pierce Biotechnology) and goat anti-human GAPDH (1:10000, Santa Cruz Biotechnologies, Santa Cruz, CA). Protein expression was visualized using an enhanced chemiluminescence detection system. Bands were identified by comparing to a molecular mass ladder (Pierce Biotechnology). The resulting blots were scanned and imported into Image J (National Institutes of Health, Bethesda, MD) in order to perform densitometry. Background was subtracted using the built-in background subtraction function in Image J to normalize the background between samples. Resulting intensity values were then normalized to the HUVEC condition for each time point. Normalized values for each condition from three separate experiments were then averaged to allow for statistical comparisons.

### Gelatin Zymography

For gelatin zymography, precast Novex zymogram gels (10% Tris-Glycine gel with 0.1% gelatin, Invitrogen, Carlsbad, CA) were loaded with 15 µg of protein per condition and separated under nonreducing conditions. The gels were then washed twice for 30 min in a 50 mM Tris-HCl (pH = 7.5), 5 mM CaCl_2_, and 2.5% Triton X-100 solution. After washing, gels were rinsed in incubation buffer (50 mM Tris-HCl (pH = 7.5), 5 mM CaCl_2_, and 1% Triton X-100) for 10 min at 37 °C with gentle agitation. The rinse was replaced with fresh incubation buffer and incubated for 20 h at 37 °C. Gels were then Coomassie stained for 1 h and destained for 15 min twice in 10% acetic acid and 40% methanol. MMP-2 and MMP-9 bands were identified by comparing to a molecular mass ladder (Pierce Biotechnology). The resulting blots were scanned and imported into Image J (National Institutes of Health, Bethesda, MD) in order to perform densitometry. Bands were processed as described previously for the Western Blot.

### Reverse Transcription and quantitative Polymerase Chain Reaction

Total RNA was purified from RIPA buffer lysed samples using the RNeasy kit (Qiagen, Valencia, CA) per manufacturer’s protocol and quantified using a Nanodrop ND-1000 (Thermo Fisher Scientific, Rochester, NY). First-strand cDNA templates were synthesized from equal amounts of total RNA for each sample using the ImProm-II Reverse Transcription System (Promega; Madison, WI), also according to manufacturer’s protocol. Quantitative PCR (qPCR) was performed using a 7500 Fast Real-Time PCR System and TaqMan Gene Expression Master Mix (Applied Biosystems, Carlsbad, CA). Predesigned qPCR primers for human MMP-2, MMP-9, MT1-MMP, and 18s rRNA were selected from the TaqMan Gene Expression Assays database (Applied Biosystems). The ΔΔCT method was used to assess the relative quantity of each target gene.

### Other reagents used

The broad spectrum MMP inhibitor BB2516 (Tocris Bioscience, Ellisville, MO) was used at an amount greater than ten-fold excess (0.1–0.2 μM) of its IC50 concentrations against MMP-2, MMP-9, and MT1-MMP^60^. The plasmin inhibitor aprotinin (Sigma-Aldrich) was used at a greater than two-fold excess (22 nM) of its IC50 concentration against plasmin. Equal volumes of dimethyl sulfoxide (DMSO, Sigma-Aldrich) were used as the vehicle control for these experiments.

### Statistical Analysis

Statistical analyses were performed using StatPlus (AnalystSoft Inc.,Walnut, CA). Data are reported as mean ± standard error of mean (SEM). One- or two-way analysis of variance (ANOVA) with a Bonferroni post-test was used to assess statistical significance between data sets. Statistical significance was assumed when p < 0.05.

### Data Availability Statement

The datasets generated during and/or analyzed during the current study are available from the corresponding author on reasonable request.

## Electronic supplementary material

Supplemental Information
