# Supplementary material for: Evaluating the potential of endothelial cells derived from human induced pluripotent stem cells to form microvascular networks in 3D cultures
Source: Sci Rep. 2018 Feb 8;8:2671. doi: 10.1038/s41598-018-20966-1 (PMC5805762; doi:10.1038/s41598-018-20966-1)
Supplement: Supplementary file 1 — Supplemental Information [file 41598_2018_20966_MOESM1_ESM.pdf]

Supplemental Information

Title: Evaluating the potential of endothelial cells derived from human induced pluripotent stem cells to form microvascular networks in 3D cultures

Authors: Jonathan R. Bezenah[1], Yen P. Kong[2], and Andrew J. Putnam[1,2,*]

Departments of [1]Chemical Engineering and [2]Biomedical Engineering, University of Michigan, Ann Arbor, Michigan, USA 48109

[*]Corresponding author:
Andrew J. Putnam, Ph.D.
Department of Biomedical Engineering
University of Michigan
2204 Lurie Biomedical Engineering Building
1101 Beal Ave
Ann Arbor, MI 48109
Phone: (734) 615-1398
Fax: (734) 647-4834
E-mail: putnam@umich.edu

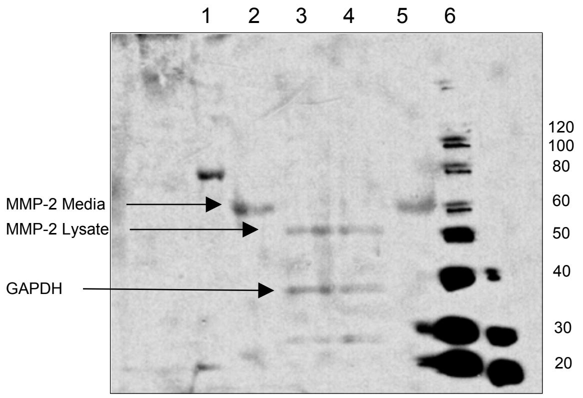

1: Kaleidoscope Protein Standard
2: HUVEC/NHLF Spent Media Day 4
3: HUVEC/NHLF Cell Lysate Day 4
4: iPSC-EC/NHLF Cell Lysate Day 4
5: iPSC-EC/NHLF Spent Media Day 4
6: Molecular Weight Marker

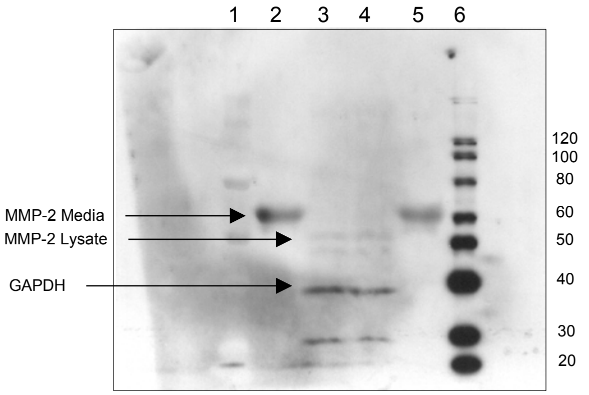

1: Kaleidoscope Protein Standard
2: HUVEC/NHLF Spent Media Day 7
3: HUVEC/NHLF Cell Lysate Day 7
4: iPSC-EC/NHLF Cell Lysate Day 7
5: iPSC-EC/NHLF Spent Media Day 7
6: Molecular Weight Marker

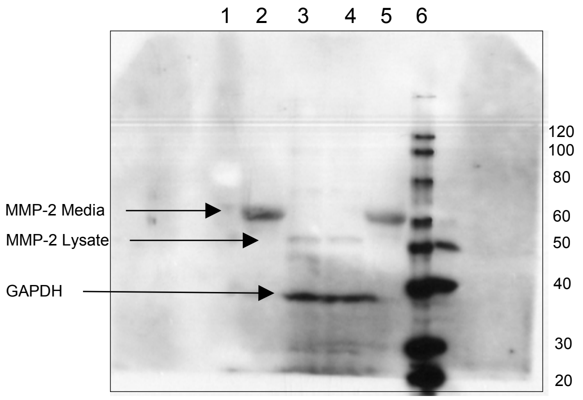

1: Kaleidoscope Protein Standard
2: HUVEC/NHLF Spent Media Day 14
3: HUVEC/NHLF Cell Lysate Day 14
4: iPSC-EC/NHLF Cell Lysate Day 14
5: iPSC-EC/NHLF Spent Media Day 14
6: Molecular Weight Marker

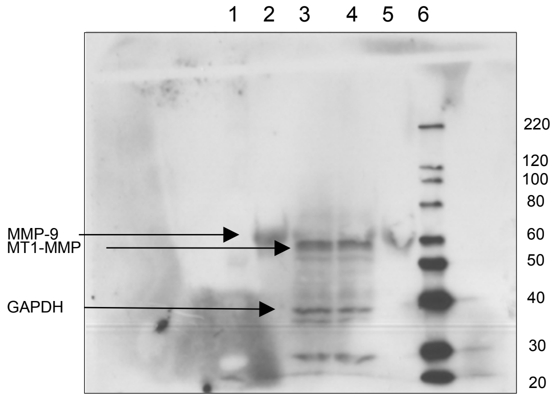

1: Kaleidoscope Protein Standard
2: HUVEC/NHLF Spent Media Day 4
3: HUVEC/NHLF Cell Lysate Day 4
4: iPSC-EC/NHLF Cell Lysate Day 4
5: iPSC-EC/NHLF Spent Media Day 4
6: Molecular Weight Marker

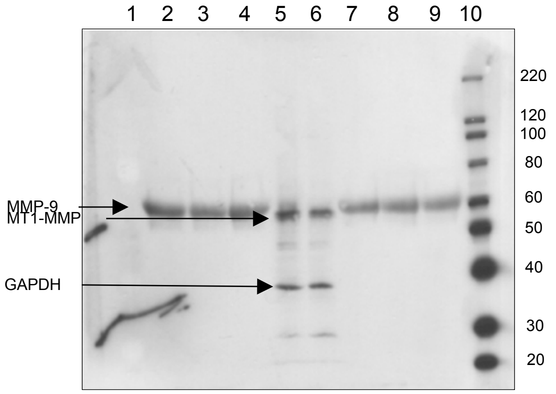

1: Kaleidoscope Protein Standard
2: HUVEC/NHLF Spent Media Day 14
3: HUVEC/NHLF Spent Media Day 4
4: HUVEC/NHLF Spent Media Day 7
5: HUVEC/NHLF Cell Lysate Day 7
6: iPSC-EC/NHLF Cell Lysate Day 7
7: iPSC-EC/NHLF Spent Media Day 7
8: iPSC-EC/NHLF Spent Media Day 4
9: iPSC-EC/NHLF Spent Media Day 14
10: Molecular Weight Marker

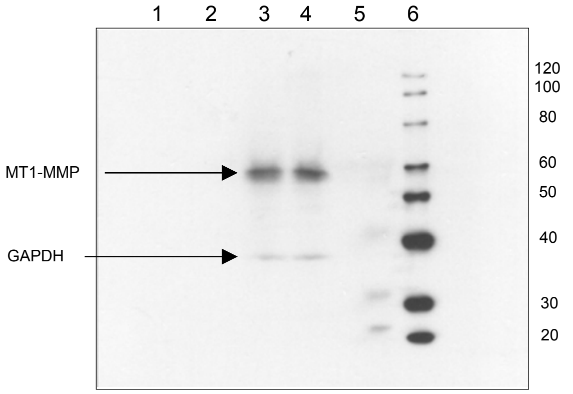

1: Kaleidoscope Protein Standard
2: HUVEC/NHLF Spent Media Day 14
3: HUVEC/NHLF Cell Lysate Day 14
4: iPSC-EC/NHLF Cell Lysate Day 14
5: iPSC-EC/NHLF Spent Media Day 14
6: Molecular Weight Marker

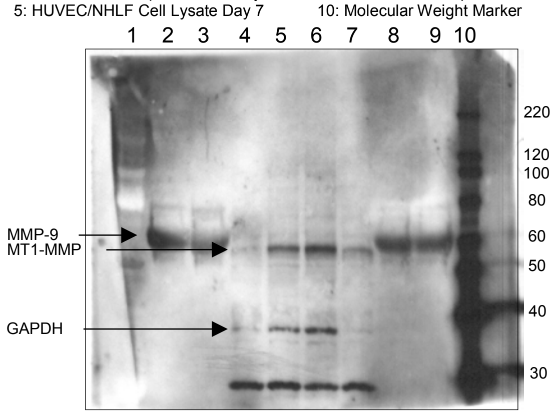

1: Kaleidoscope Protein Standard
2: HUVEC/NHLF Spent Media Day 4
3: HUVEC/NHLF Spent Media Day 7
4: HUVEC/NHLF Cell Lysate Day 4
5: HUVEC/NHLF Cell Lysate Day 7
6: iPSC-EC/NHLF Cell Lysate Day 7
7: iPSC-EC/NHLF Cell Lysate Day 4
8: iPSC-EC/NHLF Spent Media Day 7
9: iPSC-EC/NHLF Spent Media Day 4
10: Molecular Weight Marker

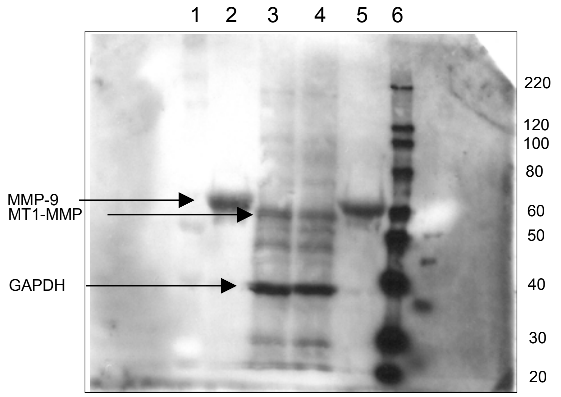

1: Kaleidoscope Protein Standard
2: HUVEC/NHLF Spent Media Day 14
3: HUVEC/NHLF Cell Lysate Day 14
4: iPSC-EC/NHLF Cell Lysate Day 14
5: iPSC-EC/NHLF Spent Media Day 14
6: Molecular Weight Marker

Supplemental Fig. S1: Full Western blot images of protein transcript levels of MMP-2, MT1-MMP, MMP-9 from HUVEC or iPSC-EC coated microcarrier beads co-cultured with NHLFS at various time points. Two protein standards were used, one for visualization during transfer and the other for visualization via chemiluminescence.

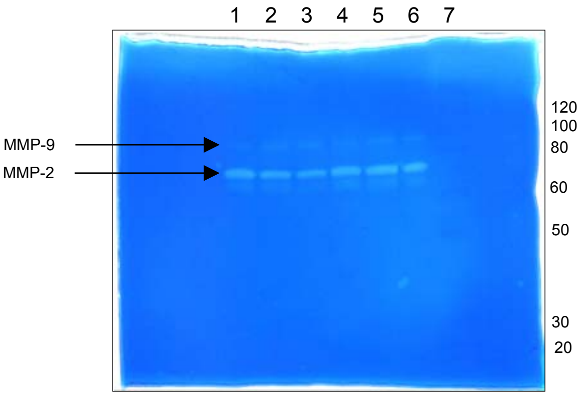

1: HUVEC/NHLF Spent Media Day 4
2: HUVEC/NHLF Spent Media Day 7
3: HUVEC/NHLF Spent Media Day 14
4: iPSC-EC/NHLF Spent Media Day 4
5: iPSC-EC/NHLF Spent Media Day 7
6: iPSC-EC/NHLF Spent Media Day 14
7. Molecular Weight Marker

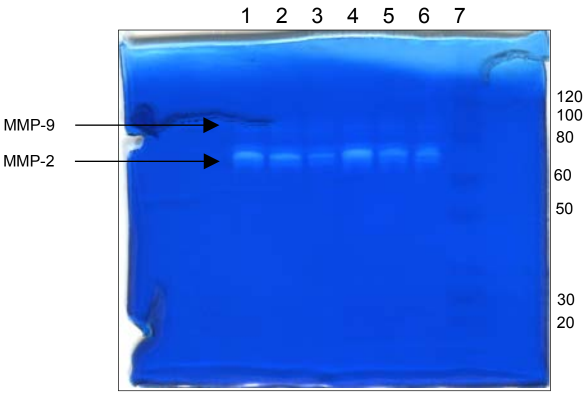

1: HUVEC/NHLF Spent Media Day 4
2: HUVEC/NHLF Spent Media Day 7
3: HUVEC/NHLF Spent Media Day 14
4: iPSC-EC/NHLF Spent Media Day 4
5: iPSC-EC/NHLF Spent Media Day 7
6: iPSC-EC/NHLF Spent Media Day 14
7. Molecular Weight Marker

Supplemental Fig. S2: Full gelatin zymography images of protein activity levels of MMP-2, MMP-9 from HUVEC or iPSC-EC coated microcarrier beads co-cultured with NHLFS at various time points.
